# Peers and teachers as the best source of social support for school engagement for both advantaged and priority education area students

**DOI:** 10.3389/fpsyg.2022.958286

**Published:** 2022-09-23

**Authors:** Delphine Martinot, Alyson Sicard, Birsen Gul, Sonya Yakimova, Anne Taillandier-Schmitt, Célia Maintenant

**Affiliations:** ^1^CNRS, Université Clermont Auvergne, Clermont-Ferrand, France; ^2^Laboratoire PAVeA (EA 2114), Université de Tours, Tours, France

**Keywords:** perceived social support, school engagement, sense of school belonging, middle school students, type of school

## Abstract

Promoting student’s school engagement is a major goal in our society. The literature has shown that students’ proximal sources of social support can play a fundamental role in facilitating this engagement. The purpose of this study was (1) to compare perceived support from four sources (mother, father, teacher, and peers) as a function of two different middle-school student backgrounds, a priority education area and a privileged area; (2) and (3) to examine the contribution of these main sources of social support, either directly or indirectly (through sense of school belonging) to school engagement; and (4) to test whether perceived social support is more strongly related to school engagement, directly or indirectly, among students from priority education school compared to students from the advantaged area. In all, 623 middle-school students (aged 11–16) from either a privileged or priority education area participated in this study. The results showed that the mother was perceived as providing more support, followed by the father, the teachers, and the peers. Students from the priority education area perceived more support from their teachers than their counterparts from the more privileged area did. A path analysis showed that each source of social support, except for maternal support, contributed to school engagement. Peers and teachers emerged as the best source of support for school engagement, having significant direct effects among students from the priority education area and both direct and indirect (through the sense of school belonging) effects among students from the advantaged area. Peer support also appears to have a double-edged effect on school engagement among students in the priority education area. This study contributes to enlightening the phenomenon of school engagement in adolescence by clarifying the role of social support and the related mediating process. Being perceived as an important source of social support by students is not enough to contribute to their sense of school belonging and school engagement.

## Introduction

Parents, educators, policymakers, and researchers share a major concern about school engagement as a key factor linked to a variety of important outcomes in the lives of youth with potentially long-term consequences. School engagement, which refers to students’ directed and sustained participation in school (Skinner and Pitzer, 2011), predicts positive academic experiences such as learning and achievement, graduation from high school, and entry into and success in higher education (Fredricks et al., 2004). It also contributes to minimizing negative outcomes, such as academic underperformance, grade retention, and school dropout (e.g., Janosz et al., 2000; Archambault et al., 2009). Decades of research have, therefore, shown great interest in the study of factors that foster the development and persistence of school engagement. Perceived social support is one of these factors (e.g., Eccles and Wigfield, 2002; Furrer and Skinner, 2003; Christenson et al., 2012; Wang and Eccles, 2012; Estell and Perdue, 2013). Research on social support in the school context has identified three main sources: parents or family (e.g., Oberle et al., 2010; Wang and Eccles, 2012), teachers (e.g., Archambault et al., 2017; Gutiérrez et al., 2017), and peers, including friends and classmates (e.g., Furrer et al., 2014). Examining which sources of social support best contribute to school engagement appears to be a fundamental step in being able to promote students’ school engagement and its positive consequences. Indeed, while there are many studies of the relationship between one or more of these proximal sources of social support and school engagement, they never examine all of the sources at once. Moreover, knowing which sources of social support contribute most to school engagement is particularly important for students at risk of being less engaged, as is the case with middle-school students. Indeed, researchers have identified the middle-school years as an especially risky period for school disengagement (e.g., Eccles et al., 1993; Eccles and Roeser, 2010; Wang and Holcombe, 2010; Wang and Fredricks, 2014). The risk of school disengagement is also increased by the negative stereotype of intellectual inferiority targeting some groups of students, such as students of low socioeconomic status or living in a deprived environment (e.g., Spencer and Castano, 2007; Désert et al., 2009; Loose et al., 2012; Martinot et al., 2020). Therefore, the purpose of the present study is to examine the relationships between the main sources of social support regarding education and school engagement among middle-school students from disadvantaged and privileged backgrounds.

### Sources of social support and school engagement

Research has clearly shown that the relationship that students have with their teachers can have a significant impact on their engagement in school (e.g., Garcia-Reid et al., 2005; Lam et al., 2012; Wang and Eccles, 2012; Estell and Perdue, 2013; Rimm-Kaufman et al., 2014; Archambault et al., 2017; Gutiérrez et al., 2017). Adolescents who have close and caring relationships with teachers presented higher school engagement (e.g., Wang and Holcombe, 2010). For instance, students perceiving high social support from their teachers are more cognitively engaged than those perceiving low support (Wang and Eccles, 2012). Perceived social support from their teachers also reduces students’ deviant and socially undesirable behaviors (Birch and Ladd, 1997). According to Christenson et al. (2012), teacher support would mainly influence behavioral engagement and cognitive engagement through instructions, academic support, and class management. In contrast, students are more behaviorally disengaged when their teachers did not respect them (Fredricks et al., 2019) or when they perceive a lack of support from teachers (e.g., Furrer et al., 2014).

Although some studies report a decrease in parental impact as adolescence progresses (Steinberg and Silverberg, 1986; Larson and Richards, 1991), other studies show that parents remain a very important source of influence throughout adolescence (Smetana et al., 2006). Parental support in the school context is defined as the extent to which parents participate in and promote their child’s education (Brewster and Bowen, 2004). Wang and Eccles (2012) identified positive association between parental support and students’ engagement in school. More precisely, parental support—through goals, expectations, monitoring, learning resources in the home, and/or academic and motivational assistance—is likely to impact adolescents’ school engagement (Wentzel, 1998; Chen, 2005; Christenson et al., 2012; Lam et al., 2012; Wang and Eccles, 2012; Im et al., 2016; Smith et al., 2020). However, research on parental involvement showed that both adolescents and parents perceived mothers to be more involved in homework/schoolwork and school functions than fathers (Paulson and Sputa, 1996). When asked to think of only one person who is supportive of their academic efforts, students usually named their mother (Newman et al., 2000). Despite the support they perceive from their mother, both boys and girls tend to model their father more (Gecas et al., 1974). Because the research presents ambiguous results and rarely compares the role of the father and mother in the child’s school engagement, it is relevant to examine how maternal and paternal support respectively contribute to academic engagement.

As young people enter adolescence, socialization through the family gradually fades in favor of peer socialization, which increasingly exerts influence on adolescents (Harris, 1998). The approval of the peer group becomes fundamental in the self-concept development during adolescence (e.g., Harter, 1988). Thus, adolescents’ relationships with their peers become closer and more intense throughout middle school (Ryan and Patrick, 2001). Middle-school students need to maintain and establish interpersonal relationships and develop social identities (Sweeting et al., 2011). Students’ school relationships would influence their engagement through shared common school values, educational expectations, attendance, aspirations for learning, and/or academic beliefs and efforts (Christenson et al., 2012; Wang and Eccles, 2012). Thus, peer support is positively related to school engagement (Chen, 2005; Juvonen et al., 2012; Wang and Eccles, 2012; Im et al., 2016; Benner et al., 2017; Wang et al., 2018). In contrast, students who have poor relationships with their peers or are actively rejected by their peers have higher levels of disengagement from school (Juvonen et al., 2012; Ladd et al., 2017). They were also more disengaged when their peers were off task (Fredricks et al., 2019). However, peer support, despite being fundamental for adolescents, might have less impact than teacher and parent support. Indeed, some studies have suggested that, compared to peer support, teacher and parent support are better predictors of student engagement and academic performance (Lam et al., 2012; Estell and Perdue, 2013). Meanwhile, there are few studies to support this argument, it is interesting to test whether peers actually contribute less than other proximal sources of social support to school engagement, especially during adolescence.

It, therefore, seems relevant to explore which source(s) of social support—teachers, parents distinguishing between father and mother, and peers—best predict school engagement, even though each of these sources is likely to predict school engagement. Moreover, researchers agree that engagement is a multidimensional construct, or a meta-construct, whose dimensions typically include behavioral, emotional, cognitive (e.g., Fredricks et al., 2004; Estell and Perdue, 2013), and more recently social engagement (Wang et al., 2019). Behavioral engagement refers to how well students behave in class, the extent of their participation in academic, social, or extracurricular activities, and the absence of disruptive behaviors, such as skipping school or getting into trouble (i.e., behavioral disengagement), (e.g., Fredricks and McColskey, 2012; Wang and Degol, 2014). Emotional engagement is defined as students’ feelings about their school, teachers, and classrooms (e.g., Estell and Perdue, 2013) and focuses on the extent of positive (and negative) reactions to teachers, classmates, academics, or the school (e.g., Fredricks and McColskey, 2012). The more negative feelings students have, the more emotionally disengaged from school. Cognitive engagement is reflected in the student’s degree of investment in learning and willingness (or unwillingness) to put in the effort necessary to understand complex ideas and master difficult skills or in his or her lack of persistence and cognitive effort to complete the task (i.e., cognitive disengagement) (e.g., Fredricks et al., 2004). Finally, social engagement is defined in terms of the degree of participation, collaboration with classmates, strengthening friendships in the school context (Linnenbrink-Garcia et al., 2011), or conversely (social disengagement), lack of interest in people at school (Wang et al., 2019). The study of engagement as multidimensional and as arising from an interaction between the student and her/his sources of social support is likely to help us better understand the complexity of students’ experiences in school and identify which social support to target more specifically in interventions.

### Sense of school belonging as a mediating process

According to the student engagement model (Christenson et al., 2012), teacher, parental, and peer support are expected to impact school engagement through the sense of school belonging. A sense of belonging or psychological membership in the school or classroom corresponds to the extent to which students feel personally accepted, respected, included, and supported by others in their school environment (Goodenow, 1993b). Students who feel a sense of belonging in an educational environment are more engaged in classroom activities, are more motivated, are more likely to participate in extracurricular activities, report a greater sense of academic self-efficacy, and experience reduced risk behavior and depressive symptoms (Goodenow, 1993a; Walton et al., 2012; Lardier et al., 2018; Aelenei et al., 2020). Conversely, a decrease in school belonging is associated with decreased academic interest, motivation, low academic achievement, and behavioral school disengagement (Goodenow, 1993a; St-Amand et al., 2017), especially in middle-school years (Benner et al., 2017). According to Benner et al. (2017), school belonging appeared to play the most prominent buffering role in relation to school engagement, and also students’ depressive symptoms or loneliness.

Sense of school belonging is achieved through the reciprocal social relationships between the student and others implied in the school context (Finn, 1989; Goodenow, 1993a). To feel a sense of belonging to their school, students must not only have confidence in their school and adopt its values but also have positive relationships with their peers and teachers (e.g., secure and satisfying social engagement; St-Amand et al., 2017). When students feel well integrated into their peer group and recognized and supported by their teachers, they will promote the values attached to the school and will be able to develop a sense of school belonging (Duru-Bellat et al., 2008), which can in turn promote student participation and, thus, plays a role in school engagement (Furrer and Skinner, 2003). On the contrary, young people who feel unsupported by school adults and classmates are at risk of developing a low or absent sense of psychological school belonging, which may reduce school engagement (Goodenow, 1993a). Thus, in the present study, we test sense of school belonging as a potential mediating process of the effect of perceived social support on school engagement in middle-school students. We hypothesize that the more social support students perceive, the higher their sense of school belonging. In turn, a higher sense of school belonging will lead to increased school engagement. We also examine whether some sources of perceived support (father, mother, teachers, and peers) are more prone to enhance students’ sense of school belonging and, consequently, their school engagement.

### Links between social support and school engagement according to students’ social background

Youth from low socioeconomic status (SES) tends to struggle more academically, typically advancing less far in school than their more affluent peers (e.g., Kena et al., 2016; Institut national de la statistique et des études économiques [INSEE], 2020). Several studies have shown that SES, parental education level, and residential neighborhood are related to disengagement from school (e.g., Kurdek and Fine, 1993; Kurdek and Sinclair, 2000). Because students from low SES and/or with less educated parents face negative stereotypes of intellectual inferiority (e.g., Spencer and Castano, 2007; Désert et al., 2009), they are likely to perceive differential treatment or social injustice because of their group membership. Such a perception can lead them to experience a higher fear of failure and uncertainty about their capacity to succeed in school (Gecas, 1989; Spencer and Castano, 2007; Désert et al., 2009; Wiederkehr et al., 2015), thereby leading them to disengage from school (e.g., Crocker et al., 1998; Major and Schmader, 1998, Major and Schmader, 2001; Major et al., 1998; Schmader et al., 2001; Martinot et al., 2020). Thus, middle-school students from disadvantaged backgrounds are at greater risk of lower school engagement than their more privileged counterparts.

However, previous studies have demonstrated the contribution that social support networks have in the lives of disadvantaged youth (Hayes et al., 2014). Recent findings suggest that social support is positively related to the sense of school membership of students from disadvantaged backgrounds (Lardier et al., 2018). Moreover, parental support has been found to increase the likelihood of school engagement in Hispanic adolescents in the United States, while teacher support has been found to have an equally beneficial effect on reducing the likelihood of school failure for these students (e.g., Brewster and Bowen, 2004; Garcia-Reid et al., 2005, Garcia-Reid, 2007). Because supportive relationships provided by parents, peers, and teachers may serve as a safety net for students evolving in a disadvantaged environment (Garcia-Reid, 2007; Garcia-Reid et al., 2015; Lardier et al., 2019), the links between social support and school engagement might be stronger for students from disadvantaged areas than for students from the more privileged areas. Moreover, research has shown that low SES students also have a more interdependently shaped self-construal than higher SES students (Stephens et al., 2014). Therefore, one objective of the present study is to compare the direct and indirect (through sense of school belonging) effects of social support on the school engagement of French students from a priority education area with those from a more privileged area. Students enrolled in French priority education middle schools come from low or very low socioeconomic backgrounds. Such schools are located in economically depressed neighborhoods and benefit from compensatory education funds. We hypothesize that because parents, peers, and teachers may serve as more of a safety net for students in the priority education areas, social support (regardless of the source) might be more beneficial for them compared to students in more privileged areas.

### Present study

The originality of this study is to examine the links among four sources of social support (father, mother, teachers, and peers), sense of school belonging, and multidimensional engagement among middle-school students from two contrasted types of school. Indeed, no study has ever examined all four sources at once to determine which source(s) students perceive as most supportive of their schooling, whether that perception is related to their social background, and whether and how (directly or indirectly) that perceived support is related to their school engagement. First, we examine which source of social support is perceived as the most supportive depending on whether students are enrolled in a priority education school—namely, students from low SES and living in a disadvantaged area—or in a school located in a more socioeconomically privileged area (i.e., students from higher SES). This question is relatively exploratory because no previous study has examined this, even though teacher support (Garcia-Reid, 2007) and paternal support (Pujol, 1995; Bardou and Oubrayrie-Roussel, 2012) could be perceived as more important for students from disadvantaged social backgrounds than for those from more privileged social backgrounds. However, given that low-SES students have a more interdependent self-construal than higher-SES students (Stephens et al., 2014), we expect that (H1) students from disadvantaged social backgrounds will perceive more social support than those from more privileged social backgrounds. Second, we explore the specific contribution of the main sources of perceived social support to school engagement. We expect that (H2) all examined sources of perceived social support (teacher, mother, father, and peer support) predict school engagement. Third, we test whether the sense of school belonging is a mediator of the effects of perceived social support on school engagement. We expect that (H3) the more social support students perceive, the higher their sense of school belonging. In turn, a higher sense of school belonging will be associated with increased school engagement. Fourth, we expect that (H4) perceived social support is more strongly related to school engagement, directly or indirectly, among students enrolled in a priority education school compared to students enrolled in a school located in a more socioeconomically privileged area.

## Materials and methods

### Participants and procedure

In all, 674 students were asked to participate in the study. A preselection was made based on their family situation and 47 students belonging to a single-parent family were not retained in order to keep only participants who rated social support from the four sources. We also excluded four participants who did respond to the teacher or peer support items, which led to a final sample of 623 participants (including 310 boys, 307 girls, and six participants who did not report their gender). The sample comprised students in sixth grade (*n* = 153), seventh grade (*n* = 186), eighth grade (*n* = 167), and ninth grade (*n* = 117). Participants ranged in age from 11 to 16 years old, with a mean of 12.97 years old (*SD* = 1.20). From a large provincial city, 323 students were enrolled in a priority education middle school. As explained earlier, French priority education middle schools are located in economically depressed neighborhoods, benefit from compensatory education funds, and enroll students from low or very low socioeconomic backgrounds. From the same city, 300 students were enrolled in a school classified as socioeconomically privileged by the Board of Education. Such classification means that students enrolled in the school come from higher socioeconomic backgrounds and live in economically advantaged neighborhoods. We do not have any data on the participants’ race or ethnicity as French legislation strictly limits the collection of such information. An institutional ethics committee approved the research protocol (#IRB00011540-2019-21). School authorities and teachers were informed of the actual purpose of the study as part of the collaboration between the first author of the manuscript and the institutions involved. Parents were informed by a letter stating the purpose of the study, the same as that given to the children on the day of the study. The study was, therefore, presented to parents and children as a survey looking at the daily life of middle-school students to learn more about them. Informed consent to participate in this study was obtained first from school authorities and teachers, then from parents, and finally from students. All were assured that the data would remain anonymous and confidential.

Participants completed a paper-and-pencil questionnaire that included the measures detailed below, which were selected to tap into the theoretical concepts. To ensure proper understanding of the items, especially for younger students, the experimenter read aloud each of the questionnaire items to all the students and then let them respond individually. At the end of the questionnaire, the experimenter debriefed the students to reveal the purpose of the study and answer any questions they had.

### Measures

#### Perceived social support

We used the Significant Other Academic Support Scale (SOASS) developed by Sands and Plunkett (2005), a 30-item scale that measures academic support from five different sources (mother, father, teachers, classmates, and close friends). Participants were asked to rate the support provided by each source on six different items using a scale ranging from 1 (*Strongly disagree*) to 7 (*Strongly agree*). Because we had no hypothesis regarding potential differences between the support provided by classmates and close friends, we considered them the same source—that is, peer support (see Supplementary material for confirmatory factor analysis). The four dimensions of support showed satisfactory reliability: mother (α = 0.86), father (α = 0.89), teacher (α = 0.88), and peer support (α = 0.91).

#### Sense of school belonging

We used a 5-item French version (Aelenei et al., 2020) of the Psychological Sense of School Membership (PSSM) Scale (Goodenow, 1993b). The participants rated each item (e.g., *I could really be myself in this class*) using a scale from 1 (*Strongly disagree*) to 7 (*Strongly agree*). The scale showed great reliability (α = 0.79) once one item was dropped because of its very low communality (0.03) and its very small correlations with the rest of the items (*r*_s_ < 0.15), which suggested it did not share much variance with the other items.

#### School engagement

Finally, participants completed the 30 items of the Multidimensional School Engagement Scale (Fredricks et al., 2019; Wang et al., 2019). As we adapted this scale to the French school context, we first conducted an exploratory factor analysis on IBM SPSS AMOS (Arbuckle, 2019) using oblimin direct rotation. To find a reliable structure for our data, we successively removed cross-loading items and items that did not significantly load on a factor (<0.30). We reached a final structure comprising 24 (out of 30) items distributed along with four factors (see Table 1). The first factor, accounting for 25.36% of the variance, included 14 items (with three reverse items) corresponding to cognitive engagement—that is, the degree of student investment in learning. The second factor included five items only describing behavioral disengagement (i.e., students’ engagement in maladaptive, anti-school behaviors) and accounted for 8.71% of the variance. The third factor comprised seven items (including three reverse items) referring to social engagement—namely, students’ daily social interaction and collaboration with peers on educational content and the development of friendships—and explained an additional 5.82% of the variance. The fourth factor comprised four items only referring to emotional disengagement (e.g., boredom, anxiety toward their school and teachers) and explained another 5.15% of the variance. Among these four factors, only three (cognitive engagement, social engagement, and behavioral disengagement) appeared to have a satisfying internal consistency (see Table 1) and were therefore retained for path analysis. Scores for each factor were calculated by computing the average of the scores for all items comprising the factor. High scores represent greater cognitive engagement, social engagement, and behavioral disengagement in the school context.

**TABLE 1:** Results from the factor analysis of school engagement.

	Factor loading				Explained variance	Cronbach’s alpha (α)
	1	2	3	4		
**Factor 1. Cognitive engagement**					25.36%	0.88
Doing well in school is important to my future	0.72					
I contribute to what we are doing in class.	0.71					
I ask questions when I don’t understand.	0.70					
I figure out what I did wrong when I make mistakes on my schoolwork.	0.69					
I keep trying even when I get stuck on my schoolwork.	0.66					
I look over my schoolwork and make sure it is done well.	0.65					
I am interested in what we are learning at school.	0.61					
I plan out how to finish my schoolwork.	0.61					
If I don’t understand a task, I give up right away.	–0.60					
I work hard in the face of difficulties at school.	0.54					
Finishing my homework fast is more important to me than doing it well.	–0.45					
I always try my best in school.	0.44					
I get involved in school activities (e.g., school events)	0.36					
I don’t pay attention in class.	–0.33					
**Factor 2. Behavioral disengagement**					8.71%	0.78
I find reasons to get out of class.		0.76				
I don’t follow school rules.		0.74				
I find ways to be late for school.		0.73				
I goof off during work time in class.		0.69				
I don’t complete my homework.		0.37				
**Factor 3. Social engagement**					5.82%	0.68
I enjoy spending time with peers at school			0.75			
I enjoy working with peers at school.			0.57			
I don’t care about the people at my school			–0.46			
Interacting with peers isn’t an important part of school for me.			–0.45			
I am open to making new friends at school.			0.43			
I enjoy working with peers at school			0.36			
I don’t have friends in school.			–0.34			
**Factor 4. Emotional disengagement**					5.15%	0.54
I feel frustrated in school.				0.64		
I feel worried in school.				0.55		
I get in trouble at school.				0.40		
I feel overwhelmed by my schoolwork.				0.39		

### Data analyses

Analyses were computed using the statistical software IBM SPSS 25 (IBM Corp, 2017) and IBM SPSS AMOS (Arbuckle, 2019). First, we conducted an ANOVA with the type of school as a between-participant factor and source of perceived social support as a within-participant factor to test our first hypothesis. Second, to test the other three hypotheses, we conducted path analysis using IBM SPSS AMOS 25 (Arbuckle, 2019) with the maximum likelihood estimation method. We performed bootstrapping (using 5,000 bootstrap samples) to anticipate potential normality issues and compute indirect effects (Hoyle, 2012). Multiple fit indices were computed to estimate the fit of the hypothesized model. Selected indices include the robust root mean square error of approximation (RMSEA; Steiger, 2016) and its 90% confidence interval, the Bentler (1990) comparative fit index (CFI), and the Tucker–Lewis Index (TLI; Tucker and Lewis, 1973). Values below 0.06 for the RMSEA and values above 0.95 for the CFI and TLI can be considered as a demonstration of a good fit between the predictive model and the data (Hu and Bentler, 1999).

## Results

### Comparison of the four sources of support

For this first objective, we conducted an ANOVA with the type of school (privileged area vs. priority education area) as a between-participant factor and social support (mother, father, teachers, and peers) as a within-participant factor to investigate which source students perceived as providing the more support and to test whether students from different socioeconomic backgrounds had different perceptions of the social support they receive. The results showed that the amount of perceived support depended on the source, *F*(3, 1863) = 332.55, *p* < 0.001, η^2^_*p*_ = 0.35. The mother (*M* = 6.16, *SE* = 0.04) was perceived as providing more support, followed by the father (*M* = 5.71, *SE* = 0.06), the teachers (*M* = 5.03, *SE* = 0.06), and the peers (*M* = 4.33, *SE* = 0.05). Thus, peers were perceived as a lesser source of academic support than the others (see Table 2). This main effect was qualified by a significant interaction with the type of school, *F*(3, 1863) = 3.08, *p* = 0.026, η^2^_*p*_ = 0.01. In both schools, the ranking from the most supportive (mother) to the least supportive (peers) sources was the same as the one presented below (see Table 2). The only significant difference between the two schools was in teacher support, which partially confirms our first hypothesis (H1). Indeed, students from the priority education area perceived more support from their teachers than students from the privileged area did (*p* < 0.01). However, the two subsamples did not differ concerning the other three sources.

**TABLE 2:** Means scores for perceived social support depending on its source and the type of school.

	Priority education school	Privileged school	*F*	*p*	τ^2^_p_
Mother	6.15 (0.06)	6.17 (0.06)	0.07	0.79	0.00
Father	5.74 (0.08)	5.69 (0.08)	0.22	0.65	0.00
Teachers	5.19 (0.08)	4.83 (0.08)	8.32	0.00	0.01
Peers	4.41 (0.07)	4.25 (0.08)	2.02	0.13	0.00

Standard errors for mean scores are presented in parentheses.

### Links between sources of social support, sense of school belonging, and school engagement

Two other main objectives of this study were to examine the links between the four sources of social support and school engagement and to investigate the mediating role of the sense of school belonging in these relationships. Thus, we conducted path analysis using the structural equation modeling software IBM SPSS AMOS (Arbuckle, 2019) to test the relationships among social support, sense of school belonging, and school engagement. It should be noted that all the effects remained significant when age and gender were included as covariates in the model.

**FIGURE 1:**
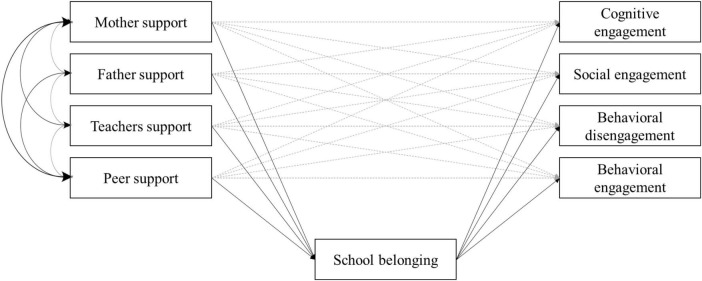
Hypothesized model regarding the relationships among social support, sense of school belonging, and school disengagement.

We developed the operational model presented in Figure 1. Correlations among all measures are presented in Table 3. All sources of social support are positively correlated with one another. Regarding the correlations between the dimensions of school engagement, the results indicated that cognitive engagement is moderately and positively related to social engagement (*r* = 0.17) but negatively related to behavioral disengagement (*r* = –0.57).

**TABLE 3:** Bivariate correlations between measures.

Variable	2	3	4	5	6	7	8	9
Mother support	0.39**	0.35**	0.24**	0.19**	0.26**	–0.13**	0.18**	0.01
Father support		0.30**	0.23**	0.18**	0.33**	–0.15**	0.150**	–0.02
Teacher support			0.42**	0.26**	0.41**	–0.26**	0.18**	–0.11**
Peer support				0.26**	0.23**	–0.03	0.37**	–0.06
School belonging					0.26**	–0.13**	0.41**	–0.14**
Cognitive engagement						–0.57**	0.17**	–0.00
Behavioral disengagement							–0.02	–0.14**
Social engagement								0.10*
School type								

*p < 0.05, **p < 0.01. School type was coded -0.5 for the priority education school and 0.5 for the privileged school.

Results of the path analysis indicated that the model did not fit the data very well, χ^2^ (3, *N* = 623) = 204.02, *p* < 0.001; RMSEA = 0.33, 90% CI [0.29,0.37]; CFI = 0.80, TLI = 0.88. The modification indices suggested that estimating the correlations between the errors of the cognitive and behavioral disengagement dimensions would significantly improve the fit of the model (MI = 172 for the correlation between cognitive engagement and behavioral disengagement). The need to estimate this correlation implies that the predictors investigated in the present model do not account for the entirety of the correlations among the two dimensions. However, they seemed to account for their correlation with the social dimension of engagement. As we only investigate predictors related to the social aspect of schooling, such an assumption makes sense. Therefore, we decided to test a new version of our model in which we added the suggested path. The results showed that this improved model fit the data very well, χ^2^ (2, *N* = 623) = 2.68, *p* = 0.26; RMSEA = 0.02, 90% CI [0.00,0.09]; CFI = 0.99, TLI = 0.99. Standardized regression coefficients for direct and indirect effects are presented in Table 4. The proportions of variance explained in the model were *R*^2^ = 0.11 for sense of school belonging, *R*^2^ = 0.23 for cognitive engagement, *R*^2^ = 0.09 for behavioral disengagement, and *R*^2^ = 0.25 for social engagement.

**TABLE 4:** Standardized coefficients, standard errors, and significance for direct and indirect effects.

	Direct effects					Indirect effects				
Variable	Estimate	SE	95% bootstrap CI		p	Estimate	SE	95% bootstrap CI		p
			LL	UL				LL	UL	
**School belonging**										
Mother support	0.07	0.05	–0.01	0.18	0.10					
Father support	0.07	0.05	–0.02	0.17	0.13					
Teacher support	0.14	0.05	0.04	0.24	0.01					
Peer support	0.17	0.04	0.08	0.26	0.00					
**Cognitive engagement**										
School belonging	0.14	0.04	0.06	0.23	0.00					
Mother support	0.05	0.04	–0.03	0.13	0.22	0.01	0.01	–0.00	0.03	0.08
Father support	0.19	0.04	0.10	0.28	0.00	0.01	0.01	–0.00	0.03	0.09
Teacher support	0.29	0.04	0.21	0.37	0.00	0.02	0.01	0.01	0.04	0.00
Peer support	0.02	0.04	–0.07	0.10	0.70	0.02	0.01	0.01	0.05	0.00
**Behavioral disengagement**										
School belonging	–0.08	0.04	–0.16	0.01	0.07					
Mother support	–0.02	0.04	–0.11	0.06	0.65	–0.01	0.01	–0.02	0.00	0.09
Father support	–0.07	0.05	–0.17	0.02	0.11	–0.01	0.01	–0.02	0.00	0.09
Teacher support	–0.27	0.05	–0.367	–0.16	0.00	–0.01	0.01	–0.03	–0.00	0.03
Peer support	0.12	0.05	0.03	0.22	0.01	–0.01	0.01	–0.03	0.00	0.04
**Social engagement**										
School belonging	0.34	0.04	0.26	0.42	0.00					
Mother support	0.06	0.04	–0.02	0.15	0.13	0.02	0.02	–0.00	0.06	0.09
Father support	0.01	0.04	–0.07	0.09	0.80	0.02	0.02	–0.01	0.06	0.12
Teacher support	–0.05	0.04	–0.14	0.03	0.23	0.05	0.02	0.01	0.09	0.01
Peer support	0.28	0.04	0.20	0.36	0.00	0.06	0.02	0.03	0.09	0.00

CI, confidence interval; LL, lower limit; UL, upper limit.

Social support appeared to have significant direct and indirect (through the sense of school belonging) effects on school engagement (see Table 4). Starting with direct effects, results showed that, as expected in H2, each source of social support, except for maternal support, predicted school engagement on at least one of these dimensions. Teacher and peer support predicted two dimensions of school engagement. Specifically, peer support was the only source to have a significant and positive effect on social engagement. It also positively predicted behavioral disengagement. Teacher support had a negative effect on behavioral disengagement and a positive effect on cognitive engagement. Cognitive engagement was also directly predicted by paternal support.

In relation to H3, the results showed that our hypothesized mediator, sense of school belonging, was predicted by the teacher and peer support and then positively predicted cognitive and social engagement and negatively behavioral disengagement. Consequently, teacher and peer support appeared to have significant indirect effects on the three dimensions of school engagement through sense of school belonging. However, social support explains a small proportion of the variance for sense of school belonging.

The fourth aim (H4) of the present study was to test whether perceived social support is more strongly related to school engagement, directly or indirectly, among students enrolled in a priority education school compared to students located in a more advantaged area. Therefore, we conducted a multiple-group analysis to test whether the paths of our model vary depending on the type of school students attend (privileged area vs. priority education area). The analysis indicated that constraining the structural weights to equality between the two groups did not lead to significant reductions in the fit of the model. However, constraining the covariances and residuals to equality between the groups led to significant reductions in the fit of the model, suggesting that these parameters differ depending on students’ background. Fit indices for the unconstrained model and model comparisons are presented in Table 5, and regression coefficients for each school type are available in Table 6.

**TABLE 5:** Model fit for the unconstrained model and model comparison.

	CMIN	DF	P	RMSEA	CFI	TLI
Unconstrained model	8.96	4	0.06	0.04	0.99	0.93
Comparison between the unconstrained model and the model containing structural weights						
	20.82	19	0.35	–0.02	–0.00	0.05
Comparison between the structural weights model and the model adding constraints to covariances						
	19.86	10	0.03	0.01	–0.01	–0.01
Comparison between the structural covariance model and the model adding constraints to residuals						
	13.10	5	0.02	0.00	–0.01	–0.01

**TABLE 6:** Standardized coefficients for direct and indirects effects depending on the type of school.

	Direct effects		Indirect effects	
	Priority education school	Privileged school	Priority education school	Privileged school
**School belonging**				
Mother support	0.13^t^	0.05		
Father support	0.02	0.12^t^		
Teacher support	0.04	0.20**		
Peer support	0.19***	0.15*		
**Cognitive engagement**				
School belonging	0.10	0.22***		
Mother support	0.05	0.03	0.02	0.01
Father support	0.22***	0.14**	0.00	0.03*
Teacher support	0.31***	0.26***	0.00	0.044***
Peer support	0.02	0.02	0.012^t^	0.034**
**Social engagement**				
School belonging	0.36***	0.36***		
Mother support	0.06	0.05	0.07*	0.02
Father support	–0.03	0.05	0.02	0.04^t^
Teacher support	0.00	–0.06	0.01	0.07**
Peer support	0.28***	0.28***	0.05**	0.05**
**Behavioral disengagement**				
School belonging	–0.07	−0.15**		
Mother support	0.04	–0.06	–0.01	–0.01
Father support	–0.07	–0.07	–0.00	−0.02*
Teacher support	−0.33***	−0.24**	–0.00	−0.03**
Peer support	0.17**	0.06	–0.01	−0.02**

^t^*p* < 0.10, *p < 0.05, ^**^p < 0.01, and *^**^p < 0.001.

Concerning the direct effects of social support on school engagement, the results showed that peer support significantly and positively predicted the social engagement for all students, but positively predicted the behavioral disengagement for students from the priority education area only. Teacher support positively predicted cognitive engagement and negatively predicted behavioral disengagement of all students. Paternal support predicted the cognitive engagement of all students. It should be noted that maternal support marginally predicted sense of school belonging for students from a priority education area only, while paternal support marginally predicted sense of school belonging for students from a privileged area only. Concerning the indirect effects, it appears that the sense of school belonging was more likely to moderate the effect of social support on the academic engagement for students from a privileged area middle school than for those from a priority education area middle school. More precisely, among students from a privileged area, teacher, peer and paternal support had an indirect effect, through sense of school belonging, on cognitive engagement and behavioral disengagement. Teacher, peer, and paternal (marginally significant) support also indirectly predicted the social engagement of these students. Among students from a priority education area, peer support had a significant indirect effect, through sense of school belonging, on both social engagement and cognitive engagement (marginally significant). Only among these students, maternal support indirectly predicted social engagement (see Table 6).

## Discussion

The purpose of this study was to examine the links among four sources of social support (father, mother, teachers, and peers), sense of school belonging, and multidimensional engagement among middle-school students from two contrasted types of school. The originality was to determine, according to the students’ social background, which source(s) the students perceived as most supportive of their schooling, whether and how (directly or indirectly) this perceived support was related to their school engagement. Therefore, (1) we compared perceived support from four sources (mother, father, teacher, and peers) as a function of two different middle-school student backgrounds, a priority education area and a privileged area; (2) and (3) we examined the contribution of these main sources of social support, either directly or indirectly (through sense of school belonging), to school engagement; and (4) we tested whether perceived social support was more strongly related to school engagement, directly or indirectly, among students from priority education school compared to students from the advantaged area.

Regarding the first objective (i.e., explore which source was perceived as the most supportive, and to compare as a function of student background), the results showed that the mother was perceived as providing more support, followed by the father, and the teachers. Peers were perceived as a lesser source of academic support than the others. Our hypothesis H1 was partially validated because students from the priority education area perceived more social support than students from the privileged area only in teacher support. In French education priority schools, the education policy gives priority to pedagogical action—specifically, coherent, caring, and demanding pedagogical and educational practices adapted to the needs of students and designed to last. These special teaching conditions seem to allow for closer and more sustained relationships between teachers and students.

Regarding the second objective (i.e., the contribution of the four sources of social support to school engagement), the results showed that our hypothesis H2 was mostly validated. Each source of social support, except for maternal support, contributed to school engagement. Perceived teacher and peer support had the strongest impact on school engagement. More precisely, the more support students perceived from their peers, the more social engagement. More surprisingly, the more support students perceived from their peers, the more behavioral disengagement they also reported. The fact that, for all adolescents, engaging in disruptive behaviors and being a “trouble-maker” can increase their popularity and prestige with their peers and give them social recognition (Coie and Jacobs, 1993; Sweeting et al., 2011) may explain such a result. Youth place increasing importance on their relationships with peers, and this priority may be in opposition to the demands of schooling (Witherspoon and Ennett, 2011). As we will explain in relation to H4, this tendency was more pronounced among students in the priority education school. However, given that social support explains only a very small proportion of the variance for behavioral disengagement, caution should be exercised in interpreting this potentially negative peer contribution.

In addition, the more students perceived that their teachers supported them, the more cognitively engaged they were and the less likely they were to behaviorally disengage from school. Such findings corroborated qualitative findings (Fredricks et al., 2019) demonstrating the important role of teachers and peers in engagement. Finally, the more support students perceived from their father, the more cognitive engagement they reported. Consistent with Gecas et al. (1974), again in 2022, students seem to model their father more when they develop beliefs about themselves (e.g., self-efficacy, motivation), and put forth the effort necessary to master difficult skills.

Consistent with H3 (and the third objective), sense of school belonging appeared to be a mediator of the effects of perceived social support on school engagement. Indeed, teacher and peer support had significant indirect effects on the social and cognitive dimensions of school engagement, through sense of school belonging. They also had a significant and negative indirect effect on behavioral disengagement. Thus, the more social support students perceived from their teacher and peers, the higher their sense of school belonging. In turn, a higher sense of school belonging predicted less disruptive behaviors. However, social support explains only a quite small proportion of variance for sense of school belonging. Even if the role of this mediating process remains modest, it seems that sense of school belonging and school engagement are promoted primarily by the social support of those who are directly involved with students in their school environment—namely, teachers and peers. In addition, although teacher and peer support directly contributed the most to the different dimensions of school engagement, students perceived them as less supportive than their parents, especially their mother. As Beckert et al. (2007) suggested, the fact that, compared to their father, most children spend more time with and have access to their mother after school probably explains why mothers are viewed more highly in most parenting domains (see also, Richardson et al., 1984; Newman et al., 2000). However, it is worth noting that the mother was the only source to not predict any dimension of school engagement. This result underscores that being perceived as an important source of social support by students is not enough to contribute to their sense of school belonging and school engagement. Therefore, it is important that researchers and education personnel do not confuse perceived or reported social support with its real contribution to school engagement.

The fourth main objective of the present study was to compare the contribution of perceived social support to school engagement as a function of student background. Perceived social support predicted school engagement for both subsamples of students. More precisely, for both student groups, paternal and teacher support positively predicted cognitive engagement, teacher support also negatively predicted behavioral disengagement. Thus, regardless of the students’ background, in predicting cognitive engagement, paternal and teacher support are positively related to students’ beliefs about themselves (e.g., self-efficacy, motivation), thinking, and willingness to put in the effort necessary to understand complex ideas and master difficult skills (e.g., Fredricks et al., 2004). In addition, teacher support is likely to reduce being off task, adopting disruptive behaviors, and/or abstaining from participation, i.e., deviant and socially undesirable behaviors related to behavioral disengagement (e.g., Birch and Ladd, 1997). By positively predicting social engagement for both subsamples, peer support is likely to improve middle-school students’ participation, collaboration with classmates, and strengthening of friendships in the school context (Linnenbrink-Garcia et al., 2011). However, peer support is likely to have a double-edged effect among students in the priority education area as it was only among these students that peer support positively predicted behavioral disengagement. On one hand, the more these students perceived social support from their peers, the more they reported social engagement. Such a result tends to show that peers may serve as a safety net for students evolving in a disadvantaged environment (Garcia-Reid, 2007; Garcia-Reid et al., 2015; Lardier et al., 2019). Feeling included by their peers would put these students in a good position to ask for help from peers (Flook et al., 2005) and, thus, can promote student participation and collaboration (Furrer and Skinner, 2003). On the other hand, the more priority education school students perceived social support from their peers, the more they reported behavioral disengagement. Such a result suggests that students from a priority education area will engage in disruptive behaviors to be accepted and appreciated by their peers. This finding is in line with previous work showing that middle-school students from a priority education area tend to develop oppositional behaviors in school to protect their social self-esteem (such as discounting their academic grades) (Martinot et al., 2020). The disadvantaged neighborhood in which these adolescents live may influence their participation in deviant behaviors (Ensminger et al., 1996) because they are more likely to associate with peers who disproportionately dropout of school compared to their counterparts from more advantaged school areas. Future studies should address which behaviors can be simultaneously perceived as high in terms of peer support (or perceived popularity) and collaboration with classmates on academic tasks as this would maximize the beneficial effect of peer support on social engagement and reduce its deleterious effect on behavioral disengagement among students from a priority education area.

In addition, the effects of perceived social support on school engagement seem to be more independent of sense of school belonging among the priority education area students compared to their advantaged counterparts. Among the priority education area students, only peer and mother support contributed to sense of school belonging and in turn, this sense was related to social engagement. Peer support was also indirectly and marginally related to cognitive engagement. Comparatively, each source of support (except for mother) is related to each dimension of engagement through sense of school belonging among students in the privileged school. Recently, Jury et al. (2019) explained the poorer sense of college belonging among low SES university students compared to their high SES counterparts by the lower prestige the former feel they have in the eyes of others. If the importance of perceived prestige in the eyes of others is already at work in adolescence, we can assume that students from a priority education area (i.e., from low SES) perceive less of it from their father and teachers than their counterparts from a privileged area. This may explain why teacher and father (more marginally) support contributed to the sense of school belonging among the latter only. However, we suggest that students from priority education backgrounds tend to feel prestige in their mother’s eyes which contributed marginally to their sense of school belonging. Future studies should explore perceived prestige among middle-school students to examine whether this perception also plays an important role in younger students’ sense of school belonging. It is worth noting that this greater impact of the sense of school belonging on school engagement among middle-school students from a privileged area is likely to constitute an important advantage in the medium and long term. Indeed, the sense of belonging predisposes students to continue to participate, even if the outcomes are not always evaluated positively (Finn, 1989).

The present research has some limitations that should be addressed. First, our study was correlational. Future experiments in which social support is induced could be helpful to address the causality issue. Second, comparing students in a priority education area to those in a more privileged area is a way of comparing students from lower socioeconomic backgrounds to those from more privileged socioeconomic backgrounds. However, there are additional confounding factors that are not controlled for, such as teacher availability, class size, and classroom environment, that are likely to impact the effect of perceived social support on school engagement beyond differences in background. Nevertheless, the present study highlights that the two student samples share more common points than differences in how social support predicts engagement with the key role of teachers and peers in engagement for all students. Third, future studies should examine whether the important role of peer support in school engagement is specific to middle school students, as the need for peer acceptance may be highest in early to mid-adolescence (e.g., Sweeting and Hunt, 2014). Fourth, the results showed that social support explains a small proportion of the variance in students’ sense of school belonging, meaning that this psychological process is probably not the best mediator to examine. Future studies should examine more cognitive mediators, such as perceived self-efficacy, a perception that may be enhanced by social support, particularly peer support (e.g., Pierce et al., 2000). The model also explains a small proportion of variance for behavioral disengagement. Because the study took place in their regular classroom, students may have been reluctant to report behaviors that they know are frowned upon by the school institution. Fifth, as expected in the literature, with our version of the French-adapted school engagement measure (Wang et al., 2019), we identified four dimensions (cognitive, behavioral, social, and emotional) from the factor analysis. The emotional and behavioral dimensions appeared exclusively as disengagement dimensions. Moreover, the emotional dimension did not have a satisfactory internal consistency. However, our exploratory factorial analysis on this scale was conducted on the same sample used in this study, which is a limitation. In the future, it will be useful to test whether these particularities are specific to France and/or related to an adolescent population by using a scale that will be validated and not simply adapted into French.

## Conclusion and perspectives

A major strength of this study was to examine the relationship between students’ four sources of proximal support and their academic engagement by considering students from contrasted backgrounds. Through this investigation, we have contributed to the literature by showing that perceived teacher and peer support is most predictive, directly or indirectly (through sense of school belonging), of school engagement for all students whether they live in a priority education area or a more privileged one. Such findings highlight that being considered the most supportive source by students is not enough to contribute to school engagement. Indeed, mothers are both the biggest and least influential source of support for students. Future studies should examine the role played by gender stereotypes in such an outcome. The gender stereotypes content (e.g., Eagly and Steffen, 1984; Eagly and Mladinic, 1994) and the motherhood myth (Ganong and Coleman, 1995; Gorman and Fritzsche, 2002) could lead people to perceive mothers as communal and caring for their children, but lacking the skills needed to guide their school engagement.

Given that adolescents spend much of their time at school, where relationships with both teachers and other students matter for development (Crosnoe and Benner, 2015), and that it is more difficult to act on the parents from the educational system, the greater contributions of teacher and peer support to school engagement are encouraging avenues for action from education professionals. This suggests that middle schools can capitalize on social support networks, including peer groups and teachers. Through these social networks, they could promote a sense of belonging and a learning environment that is safe and encouraging. In other words, all students, especially those from disadvantaged backgrounds, could improve their school engagement if their teachers and educational staff strive to create or reinforce social support that reframes students’ role identities in terms of cooperation with each other and with the teacher. This active implication of school members can generate a virtuous circle in the development of students’ school engagement. According to Furrer et al. (2014), instrumentally supportive interactions between classmates (e.g., interpreting teacher instructions and sharing materials) promote feelings of competence and autonomy through understanding each other’s viewpoints. Increasing the level of peer support is likely to improve the sense of school belonging, especially among students from disadvantaged areas, both factors are beneficial for middle-school students as they make the transition to high school (Benner et al., 2017).

Finally, the present results allow for a discussion of the potential harms of distance education. The COVID-19 pandemic has affected educational systems worldwide, leading to the near-total closures of schools, universities, and colleges. The distance learning programs that teachers could use to reach learners remotely and limit the disruption of education were probably largely insufficient to maintain the relationships between peers and minimalists in terms of perceived teacher support.

## Data availability statement

The datasets presented in this study can be found in online repositories. The names of the repository/repositories and accession number(s) can be found below: https://osf.io/c3rgt/?view_only=1383f93211064744aea4e0ed05056160.

## Ethics statement

The studies involving human participants were reviewed and approved by the Comité d’éthique de la recherche IRB-UCA: IRB00011540-2019-21. Written informed consent to participate in this study was provided by the participants’ legal guardian/next of kin.

## Author contributions

DM contributed to the supervision, conceptualization, methodology, writing of the first draft of the manuscript, and funding acquisition. AS contributed to the writing and performed the statistical analyses. BG contributed to the conceptualization and methodology and collected the data. SY participated to the statistical analyses. AT-S and CM contributed to the manuscript revision. All authors read and approved the submitted version.
