# Supplementary material for: Peers and teachers as the best source of social support for school engagement for both advantaged and priority education area students
Source: Front Psychol. 2022 Sep 23;13:958286. doi: 10.3389/fpsyg.2022.958286 (PMC9537635; doi:10.3389/fpsyg.2022.958286)
Supplement: Supplementary file 1 [file Table_1.DOCX]

**Table X**

*Confirmatory analysis of academic support*

|  | Factor loading | | | | Percentage of explained variance |
| --- | --- | --- | --- | --- | --- |
|  | 1 | 2 | 3 | 4 |  |
| **Factor 1. Peer’s support** | | | | | 29.12% |
| Friends_3 | .761 |  |  |  |  |
| Friends_5 | .743 |  |  |  |  |
| Friends_1 | .722 |  |  |  |  |
| Friends_4 | .700 |  |  |  |  |
| Friends_6 | .674 |  |  |  |  |
| Classmates_3 | .673 |  |  |  |  |
| Classmates_4 | .661 |  |  |  |  |
| Classmates_5 | .644 |  |  |  |  |
| Classmates_1 | .644 |  |  |  |  |
| Friends_2 | .606 |  |  |  |  |
| Classmates_6 | .571 |  |  |  |  |
| Classmates_2 | .511 |  |  |  |  |
| **Factor 2. Father’s support** | | | | | 13.57% |
| Father_5 |  | .898 |  |  |  |
| Father_6 |  | .848 |  |  |  |
| Father_1 |  | .784 |  |  |  |
| Father_3 |  | .729 |  |  |  |
| Father_2 |  | .668 |  |  |  |
| Father_4 |  | .643 |  |  |  |
| **Factor 3. Teacher’s support** | | | | | 8.33% |
| Teacher_4 |  |  | .810 |  |  |
| Teacher_6 |  |  | .774 |  |  |
| Teacher_5 |  |  | .749 |  |  |
| Teacher_1 |  |  | .672 |  |  |
| Teacher_3 |  |  | .669 |  |  |
| Teacher_2 |  |  | .605 |  |  |
| **Factor 4. Mother’s support** | | | | | 7.37% |
| Mother_1 |  |  |  | .778 |  |
| Mother_3 |  |  |  | .761 |  |
| Mother_5 |  |  |  | .741 |  |
| Mother_6 |  |  |  | .653 |  |
| Mother_4 |  |  |  | .631 |  |
| Mother_2 |  |  |  | .619 |  |

*Note.* N = 623. Only factor loadings > .30 are reported. Item 1 = This person helps me to do well in school, item 2 = This person motivates me to remain in school, item 3 = This person is important to help me do my schoolwork, item 4 = This person encourages me to continue my education beyond middle school, item 5 = This person gives me good advice about my education, item 6 = This person is interested in my education.
